# Validation of the efficacy of the GPSS questionnaire for screening obstructive sleep apnea

**DOI:** 10.3389/fmed.2025.1645703

**Published:** 2025-10-16

**Authors:** Zhuoji Li, Hanyue Liu, Wang Liu, Shuyue Zhou, Xiaomi Chen, Huimin Chen, Qinghua Chen, Siyu He, Zhitao Miao, Junfen Cheng, Zhaojun Chen, Yuli Cai, Huizhao Liao, Tingting Sun, Riken Chen, Lijuan Zeng, Lishu Zhang

**Affiliations:** ^1^The Second Affiliated Hospital of Guangdong Medical University, Zhanjiang, Guangdong, China; ^2^Guangdong Pharmaceutical University, Guangzhou, Guangdong, China; ^3^State Key Laboratory of Respiratory Disease, National Clinical Research Center for Respiratory Disease, Guangzhou Institute of Respiratory Health, The First Affiliated Hospital of Guangzhou Medical University, Guangzhou, Guangdong, China

**Keywords:** obstructive sleep apnea, GPSS questionnaire, diagnostic efficacy, NoSAS, STOP-bang

## Abstract

**Objective:**

This study aimed to compare the screening performance of a newly developed questionnaire for obstructive sleep apnea (OSA), the General Practice Sleep Scale (GPSS), with four commonly used screening questionnaires (NoSAS, Berlin, STOP, and STOP-Bang) across different levels of OSA severity, in order to assess their applicability in clinical practice.

**Methods:**

The study retrospectively included 2,169 patients from the Sleep Medicine Center of the First Affiliated Hospital of Guangzhou Medical University (January 2012 to June 2017) as the first group, and 310 patients from the Sleep Center of the Second Affiliated Hospital of Guangdong Medical University (January 2020 to June 2025) as the second group, all of whom were assessed for OSA. The sensitivity, specificity, positive predictive value, negative predictive value, and area under the curve (AUC) of each OSA screening questionnaire were calculated to evaluate their diagnostic performance.

**Results:**

The prevalence of OSA was 69.3% in the first group and 79.5% in the second group. In the first group, neck circumference, waist circumference, pulse rate, systolic blood pressure, diastolic blood pressure, age, height, weight, BMI, and sex showed significant differences between patients with OSA and those without OSA. In the comparison of sensitivity, specificity, PPV, and NPV of the five questionnaires among patients in the first group: for all OSA patients, the GPSS questionnaire showed higher specificity and positive predictive value compared to the other questionnaires; however, for moderate/severe OSA patients, the GPSS questionnaire demonstrated lower sensitivity, specificity, PPV, and NPV than the other four questionnaires. In the second group, among all OSA patients, the GPSS questionnaire demonstrated higher sensitivity, positive predictive value, and negative predictive value compared to the other questionnaires; among moderate/severe OSA patients, the GPSS questionnaire showed higher specificity and positive predictive value compared to the other questionnaires. ROC analysis results showed that, in the first group, the GPSS questionnaire demonstrated superior sensitivity, specificity, positive predictive value, and negative predictive value compared to the other questionnaires among all OSA patients, moderate OSA patients, and severe OSA patients. The corresponding AUC values were 0.75 (95% CI: 0.73–0.77), 0.73 (95% CI: 0.71–0.75), and 0.73 (95% CI: 0.71–0.75), respectively. In the second group, the GPSS questionnaire outperformed the other four scales in all OSA patients and moderate OSA patients, with AUC values of 0.77 (95% CI: 0.72–0.83) and 0.75 (95% CI: 0.70–0.80), respectively. However, it still had some reference value in patients with severe OSA, with a value of 0.69 (95% CI: 0.62–0.75).

**Conclusion:**

According to the ROC results, the GPSS questionnaire was superior to the other four scales in screening for mild-to-moderate OSA, though there remains room for improvement in detecting severe OSA. Therefore, we recommend the GPSS for early screening of OSA, especially in primary healthcare settings with limited time. For patients at high risk of severe OSA, a combination of GPSS and other screening tools could be considered.

## 1 Background

Obstructive sleep apnea (OSA) is characterized by repeated collapse of the upper airway during sleep (1). OSA is a common condition affecting up to 20% of adults (2). OSA is known to reduce quality of life and is associated with several common comorbidities, such as coronary artery disease, hypertension, cerebrovascular accidents, gastroesophageal reflux disease, congestive heart failure, and myocardial infarction (3–5). The average life expectancy of untreated OSA patients is estimated to be 58 years, 20 years shorter than the average life expectancy of the general population (6).

The current gold standard for the diagnosis of OSA is polysomnography (PSG) (7), and the severity level of sleep apnea (OSA) is measured by the Apnea Hypopnea Index (AHI). However, the test must be performed in a specialized sleep center in the presence of a trained professional, which is time-consuming, labor-intensive, and expensive. Despite increasing awareness of the condition and improved diagnostic modalities, there are barriers to accessing PSG due to availability or financial constraints (8). Therefore, PSG is not easily accessible in primary care. The majority of patients remain undiagnosed and untreated (9), and in China, approximately 84–93% of patients with clinically significant OSA fail to receive a timely diagnosis (10), especially in remote areas (11). This situation prompts an urgent need for the development of a tool that is widely applicable at the grassroots level and capable of performing early screening for OSA in the general population. An ideal screening tool should have feasibility and considerable accuracy (12). To date, scholars in various countries have developed a variety of OSA screening tools, and some of these scales are complicated and require the use of computers and complex mathematical calculations, which makes it difficult to promote their use in clinical settings (10).

The General Practice Sleep Scale (GPSS) was proposed by Howarth et al. (13) in Australia. The GPSS questionnaire is a simple nine-question OSA risk assessment tool, which can be adopted and used in the general practice (GP) setting. GPs often encounter patients with complex medical problems in their daily clinical practice, and due to time constraints, they are unable to implement any of the multiple or complex OSA screening tools (14, 15). A simplified sleep screening tool, such as the GPSS questionnaire, may be more expedient. By creating synergy between the patient and the primary healthcare provider, the tool can be completed by the patient in the waiting room, thus triggering further investigation of the presence of OSA in high-risk patients.

This study aimed to compare the screening performance of a newly developed screening questionnaire for OSA (General Practice Sleep Scale, GPSS) and four commonly used screening questionnaires (NoSAS, Berlin, STOP, and STOP-Bang) at different OSA severity levels to assess their applicability in clinical practice.

## 2 Materials and methods

### 2.1 Data source

The study retrospectively included 2,169 patients from the Sleep Medicine Center of the First Affiliated Hospital of Guangzhou Medical University (January 2012 to June 2017) as the first group and 310 patients from the Sleep Center of the Second Affiliated Hospital of Guangdong Medical University (January 2020 to June 2025) as the second group, after obtaining the approval of the Ethics Committee of the First Affiliated Hospital of Guangzhou Medical University and the Second Affiliated Hospital of Guangdong Medical University (2,022,183, PJKT2024-050). We analyzed data collected from face-to-face interviews during previous visits.

### 2.2 Information collection

Basic information and questionnaires of patients were collected: (1) basic anthropological information: age, sex, occupation, education; (2) anthropometric indicators: height, weight, neck circumference, waist circumference; (3) past history: history of hypertension, diabetes mellitus, cardio-cerebral vascular diseases, ear, nose and throat diseases and other related medical history; (4) personal history: history of smoking and alcohol consumption; (5) nighttime sleep: respiratory-related symptoms during sleep, such as snoring, apnea, and wakefulness, with their severity level and duration assessed separately for OSA.

According to the guidelines for the diagnosis and treatment of OSA (16), OSA was determined by an apnea-hypopnea index (AHI) ≥ 5 episodes/h. The condition was graded as follows: normal group (5 episodes/h), mild OSA group (5–15 episodes/h), moderate OSA group (15–30 episodes/h), and severe OSA group (≥30 episodes/h).

### 2.3 Screening questionnaire

1. GPSS (13): The GPSS ranges from 0 to 22 points and includes 9 questions: ① sex: male = 2 points; ② body mass index (BMI): >25 kg/m^2^ and <30 kg/m^2^ = 1 point, ≥30 kg/m^2^and < 40 kg/m^2^ = 2 points, ≥40 kg/m^2^ = 5 points; ③ age >34 years and ≤45 years = 1 point, >45 years = 3 points; ④ neck circumference >39.5 cm and ≤46 cm for men, >35.5 and ≤38 cm for women = 4 points; male >46 cm, female >38 cm = 5 points; ⑤ snoring = 3 points; ⑥ apnea = 1 point; ⑦ wake up unrefreshed = 1 point; ⑧ daytime sleepiness = 1 point; ⑨ comorbidities (hypertension, depression, diabetes mellitus, heart disease) = 1 point. A GPSS score of ≥7 points indicates a risk of OSA.
2. NoSAS (17): The NoSAS score ranges from 0 to 17 points and includes 5 questions: ① Neck circumference >40 cm = 4 points; ② Body mass index (BMI): ≥25 kg/m^2^ and <30 kg/m^2^ = 3 points, and ≥30 kg/m^2^ is 5 points; ③ Snoring = 2 points; ④ Age ≥55 years = 4 points; ⑤ Sex: male = 2 points. A NoSAS score ≥8 points indicates a risk of OSA.
3. STOP questionnaire (18): The STOP score ranges from 0 to 4 points, including 4 questions: snoring, fatigue, observed apnea, and hypertension. Answer with ‘yes ‘or’ no, ‘yes’ = 1 point, ‘no’ = 0 points. If the score of the 4 questions is > 2 points, it is classified as high risk of OSA. If the score of 4 questions >2, it is considered a risk of OSA.
4. STOP-Bang questionnaire The STOP-Bang questionnaire is based on the STOP questionnaire with 4 additional items: body mass index >35 kg/m^2^, age >50 years, neck circumference >40 cm, and male, and each item is scored as 1 point for ‘yes’ and 0 points for ‘no’. The 8-item ‘yes’ is 1 point, and ‘no’ is 0 points. ‘Yes’ for each item is 1 point, ‘No’ is 0 points, and a score of ≥3 for 8 questions indicates a risk of OSA.
5. Berlin questionnaire (19): The questionnaire consists of 11 questions divided into 3 groups: ① severity level of snoring; ② daytime sleepiness; ③ hypertension or obesity. The scores of each group were calculated and categorized as either negative or positive. If ≥2 of the 3 groups were positive, the patient was considered to be at high risk of OSA (high-risk group), and if only 1 or no group was positive, the patient was considered to be at low risk of apnea (low-risk group).

### 2.4 Statistical analyses

**Figure 1:**
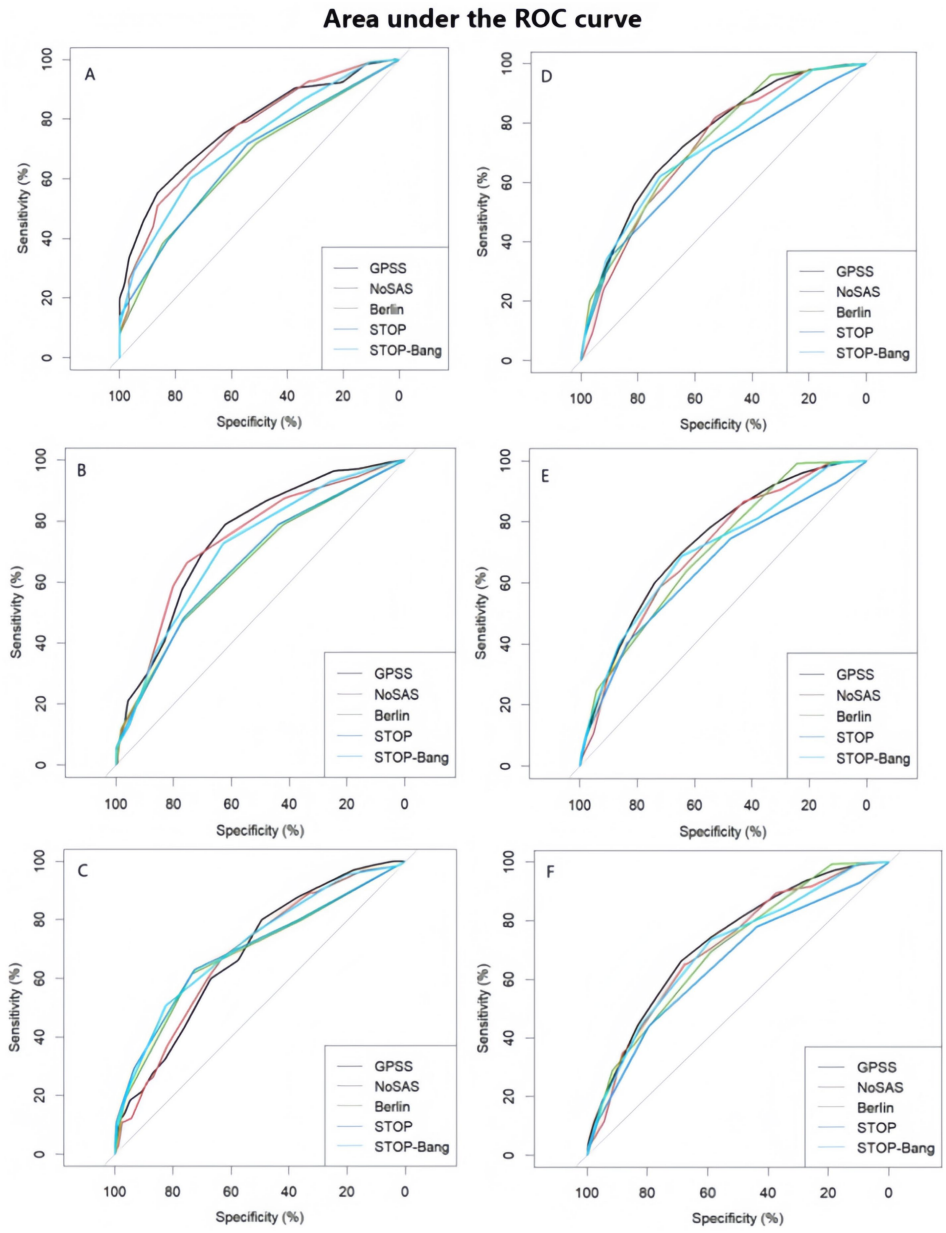
Performance and comparison of five types of questionnaires **(A)** Area under the ROC curve (AUC) of the five scale scores of the first group (AHI = 5 as cut-off point) **(B)** Area under the ROC curve (AUC) of the five scale scores of the first group (AHI = 15 is the cut-off point). **(C)** Area under the ROC curve (AUC) for the five scale scores of the first group (AHI = 30 is the cut-off point). **(D)** Area under the ROC curve (AUC) of the five scale scores of the second group (AHI = 5 as cut-off point) **(E)** Area under the ROC curve (AUC) for the five scale scores of the second group (AHI = 15 is the cut-off point). **(F)** Area under the ROC curve (AUC) for the five scale scores in the second group (AHI = 30 as cut-off point).

Statistical analyses were performed using the IBM SPSS Statistics 26 program. Categorical variables were described by frequencies and percentages, while quantitative variables were described by means, medians, standard deviations, maximum, and minimum values. The F-test is used to determine whether there are significant differences in continuous variables (such as age, weight, and BMI) across different groups. The chi-squared test is used to analyze whether there are significant differences in categorical variables (such as sex distribution) across different groups. When the *p*-value is 0.001, it can be calculated that there is a significant difference in GPSS scores between different OSA severity level groups, indicating a possible association between GPSS scores and OSA severity levels (Figure 1).

To assess the performance of GPSS scores in predicting OSA, sensitivity, specificity, negative predictive values (NPVs), and positive predictive values (PPVs) were estimated for the different AHI cut-off values. The diagnostic efficacy of each questionnaire at different AHI (5, 15, and 30 episodes/h) thresholds was evaluated by several indicators [sensitivity, specificity, positive predictive value, negative predictive value, and area under the ROC curve (AUC)] and their respective 95% confidence intervals (CI).

In order to measure the diagnostic ability of the GPSS scores for different AHI thresholds, we also determined the area under the subject’s work characteristics (ROC) curve, calculated the sensitivity, specificity, positive predictive value, and negative predictive value of the five scales, and reported their respective 95% confidence intervals (CI) and diagnostic odds ratios (DORs) in order to assess the diagnostic value of the five screening scales for OSA.

## 3 Results

### 3.1 Comparison of general information

Of the 2,169 patients in the first group, 78.1% were male, with a mean age of 47.6 ± 13.9 years, an average neck circumference of 38.4 ± 3.9 cm, and a mean BMI of 26.4 **±** 4.1 kg/m^2^. OSA was present in 69.3% of the patients, of which 30.3% had mild OSA, 23.2% had moderate OSA, and 46.5% had severe OSA. Of the 310 patients in the second group, 83.2% were male, with a mean age of 66.1 ± 18.8 years. The average neck circumference was 36.3 ± 4.6 cm, and the mean BMI was 23.5 ± 4.8 kg/m^2^. OSA was present in 79.5% of the patients, of whom 33.2% had mild OSA, 25.5% had moderate OSA, and 41.3% had severe OSA. Table 1 shows descriptive summary statistics and comparisons between the OSA and non-OSA groups. The results show that neck circumference, waist circumference, pulse, systolic blood pressure, diastolic blood pressure, age, height, weight, BMI, and sex characteristics were significantly different between OSA and non-OSA groups.

**Table 1:** Summary statistics of the patient population and baseline comparisons between the OSA and non-OSA groups.

Variables	Total	Normal Group	Mild OSA	Moderate OSA	Severe OSA	Statistic	*p*
Number of cases	2,169	664	458	347	700	–	–
GPSS, M (Q₁, Q₃)	11.00 (8.00, 14.00)	8.00 (6.00,11.00)	10.00 (8.00,13.00)	11.00 (9.00,14.00)	13.00 (10.00,15.00)	*χ*^2^ = 456.58	<0.001
NoSAS, M (Q₁, Q₃)	8.00 (6.00, 11.00)	6.00 (4.00,9.00)	8.00 (6.00,11.00)	9.00 (7.00,11.00)	11.00 (8.00,13.00)	*χ*^2^ = 367.78	<0.001
Berlin, M (Q₁, Q₃)	2.00 (1.00, 2.00)	1.00 (0.00,2.00)	2.00 (1.00,2.00)	2.00 (1.00,2.00)	2.00 (1.00,3.00)	*χ*^2^ = 411.14	<0.001
STOP, M (Q₁, Q₃)	2.00 (1.00, 3.00)	1.00 (1.00,2.00)	2.00 (1.00,2.00)	2.00 (1.00,3.00)	2.00 (2.00,3.00)	*χ*^2^ = 224.04	<0.001
STOP-Bang, M (Q₁, Q₃)	4.00 (2.00, 5.00)	3.00 (2.00,4.00)	3.00 (2.00,4.00)	4.00 (2.50,5.00)	4.00 (3.00,5.00)	*χ*^2^ = 363.70	<0.001
Neck circumference (cm)	38.38 ± 3.93	36.40 ± 3.86	37.93 ± 3.59	38.64 ± 3.32	40.42 ± 3.45	*F* = 145.31	<0.001
Waist circumference (cm)	95.16 ± 11.39	89.69 ± 11.02	93.85 ± 10.07	96.00 ± 10.18	100.79 ± 10.38	*F* = 130.63	<0.001
Pulse (beats per minute)	80.27 ± 13.42	79.89 ± 14.09	78.17 ± 12.20	78.80 ± 13.06	82.80 ± 13.35	*F* = 12.69	<0.001
Systolic blood pressure (mmHg)	134.90 ± 18.52	125.05 ± 16.98	134.60 ± 16.77	137.59 ± 17.82	143.08 ± 16.91	*F* = 128.75	<0.001
Diastolic blood pressure (mmHg)	83.12 ± 12.30	78.29 ± 11.11	82.08 ± 11.45	83.41 ± 11.69	88.21 ± 12.23	*F* = 83.08	<0.001
Age (years)	47.60 ± 13.88	47.08 ± 14.73	49.67 ± 13.14	49.70 ± 14.10	45.70 ± 13.11	*F* = 10.91	<0.001
Height (cm)	166.53 ± 7.75	164.71 ± 7.93	165.57 ± 7.74	166.85 ± 8.00	168.72 ± 6.87	*F* = 34.90	<0.001
Weight (kg)	73.60 ± 13.81	67.42 ± 13.07	71.45 ± 11.84	74.03 ± 12.33	80.64 ± 13.18	*F* = 127.94	<0.001
BMI (kg/m^2^)	26.44 ± 4.08	24.76 ± 3.98	25.99 ± 3.50	26.55 ± 3.70	28.28 ± 3.94	*F* = 98.66	<0.001
Sex n (%)						*χ*^2^ = 134.96	<0.001
Male	1,694 (78.10)	436 (65.66)	342 (74.67)	277 (79.83)	639 (91.29)		
Female	475 (21.90)	228 (34.34)	116 (25.33)	70 (20.17)	61 (8.71)		

### 3.2 Diagnostic efficacy of the GPSS questionnaire

With a GPSS score of ≥8, the sensitivity for all OSA was 88.6%, with a PPV of 77.4% and a NPV of 61.8%; for moderate OSA, the sensitivity was 92.0%, with a PPV of 56.0% and a NPV of 81.3%; and for severe OSA, the sensitivity was 93.7%, with a PPV of 38.0% and NPV of 90.2%. The specificity and PPV gradually increased as the score increased from 8 to 12. Table 2 summarizes the sensitivity, specificity, PPV, and NPV for all OSA, moderate OSA, and severe OSA.

**Table 2:** Predictive parameters for each GPSS score cut-off at different AHI levels.

All OSA (AHI > 5)					
GPSS score cut-off	*N* (%)	Sensitivity	Specificity	PPV	NPV
≥0	1,503 (69.3)	100	0.0	69.3	-
≥1	1,502 (69.2)	99.9	0.3	69.3	66.7
≥2	1,502 (69.2)	99.9	1.2	69.5	88.9
≥3	1,502 (69.2)	99.9	3.0	69.9	95.2
≥4	1,499 (69.1)	99.7	7.1	70.8	92.2
≥5	1,489 (68.6)	99.1	11.9	71.7	84.9
≥6	1,470 (67.8)	97.8	20.1	73.4	80.2
≥7	1,422 (65.6)	94.6	31.2	75.6	72.0
≥8	1,331 (61.4)	88.6	41.7	77.4	61.8
≥9	1,218 (56.2)	81.0	52.4	79.3	55.0
≥10	1,087 (50.1)	72.3	64.1	82.0	50.7
≥11	947 (43.7)	63.0	73.9	84.5	46.9
≥12	794 (36.6)	52.8	81.2	86.4	43.3
≥13	634 (29.2)	42.2	86.2	87.3	39.8
≥14	493 (22.7)	32.8	90.8	89.0	37.5
≥15	323 (14.9)	21.5	94.9	90.5	34.9
≥16	192 (8.9)	12.8	97.6	92.3	33.1
≥17	97 (4.5)	6.5	99.1	94.2	31.9
≥18	30 (1.4)	2.0	99.8	96.8	31.1
≥19	7 (0.3)	0.5	100	100	30.8
≥20	1 (0.05)	0.1	100	100	30.7
21	0 (0.0)	0.0	100	-	30.7

Moderate/severe OSA (AHI > 15)					
GPSS score cut-off	*N* (%)	Sensitivity	Specificity	PPV	NPV
≥0	1,047 (48.3)	100	0.0	48.3	-
≥1	1,047 (48.3)	100	0.3	48.3	100
≥2	1,047 (48.3)	100	0.8	48.5	100
≥3	1,047 (48.3)	100	1.9	48.7	100
≥4	1,046 (48.2)	99.9	4.5	49.4	98.0
≥5	1,043 (48.1)	99.6	7.9	50.2	95.7
≥6	1,030 (47.5)	98.4	13.4	51.4	89.8
≥7	1,007 (46.4)	96.2	22.2	53.6	86.2
≥8	963 (44.4)	92.0	32.6	56.0	81.3
≥9	900 (41.5)	86.0	43.4	58.6	76.8
≥10	818 (37.7)	78.1	54.7	61.7	72.8
≥11	728 (33.6)	69.5	65.0	64.9	69.6
≥12	628 (29.0)	60.0	74.1	68.3	66.5
≥13	512 (23.6)	48.9	80.9	70.5	62.9
≥14	403 (18.6)	38.5	86.5	72.7	60.1
≥15	269 (12.4)	25.7	92.2	75.4	57.1
≥16	161 (7.4)	15.4	95.8	77.4	54.8
≥17	84 (3.9)	8.0	98.3	81.6	53.4
≥18	28 (1.3)	2.7	99.7	90.3	52.3
≥19	7 (0.3)	0.7	100	100	51.9
≥20	1 (0.0)	0.1	100	100	51.8
21	0 (0.0)	0.0	100	-	51.7

Severe OSA (AHI > 30)					
GPSS score cut-off	N (%)	Sensitivity	Specificity	PPV	NPV
≥0	698 (32.2)	100	0.0	47.5	-
≥1	698 (32.2)	100	0.2	32.2	100
≥2	698 (32.2)	100	0.6	32.3	100
≥3	698 (32.2)	100	1.4	32.5	100
≥4	697 (32.1)	99.9	3.4	32.9	98.0
≥5	696 (32.1)	99.7	6.2	33.5	97.8
≥6	690 (31.8)	98.9	10.8	34.5	95.2
≥7	678 (31.3)	97.1	18.3	36.1	93.1
≥8	654 (30.2)	93.7	27.6	38.0	90.2
≥9	617 (28.4)	88.4	37.6	40.2	87.2
≥10	569 (26.2)	81.5	48.5	42.9	84.7
≥11	519 (23.9)	74.4	59.1	46.3	82.9
≥12	462 (21.3)	66.2	68.9	50.3	81.1
≥13	382 (17.6)	54.7	76.6	52.6	78.1
≥14	309 (14.2)	44.3	83.3	55.8	75.9
≥15	208 (9.6)	29.8	89.9	58.3	73.0
≥16	132 (6.1)	18.9	94.8	63.5	71.1
≥17	74 (3.4)	10.6	98.0	71.8	69.8
≥18	26 (1.2)	3.7	99.7	83.9	68.6
≥19	6 (0.3)	0.9	99.9	85.7	68.0
≥20	1 (0.0)	0.1	100	100	67.9
21	0 (0.0)	0.0	100	-	67.8

In the comparison of sensitivity, specificity, PPV and NPV of the five questionnaires in the first group of patients, in all OSA patients, the GPSS questionnaire was superior to the other questionnaires in terms of specificity and positive predictive value; and in patients with moderate/severe OSA, the GPSS questionnaire was inferior to the remaining four questionnaires in terms of sensitivity, specificity, PPV and NPV. In the second group, in all OSA patients, the GPSS questionnaire was superior to the other questionnaires only in terms of sensitivity, positive predictive value, and negative predictive value; in patients with moderate/severe OSA, the GPSS questionnaire was superior to the other questionnaires in terms of specificity and positive predictive value. Therefore, it is not possible to judge the superiority of the questionnaire from individual indicators. The sensitivity, specificity, positive predictive value, negative predictive value, and DOR of different degrees of OSA in the first and second groups are shown in Tables 3, 4.

**Table 3:** Different AHIs as cut-off points for the diagnosis of obstructive sleep apnea (first group).

Questionnaire	ROC	Sensitivity	Specificity	Positive Predictive Value	Negative Predictive Value	Diagnostic Odds Ratios
AHI = 5						
GPSS	0.75(95% CI:0.73 ~ 0.77)	0.89(95% CI:0.87 ~ 0.90)	0.42(95% CI:0.38 ~ 0.46)	0.77 (95% CI:0.76 ~ 0.79)	0.62 (95% CI:0.57 ~ 0.66)	5.9 (95% CI:4.7 ~ 7.3)
NoSAS	0.72 (95% CI:0.70 ~ 0.74)	0.88 (95% CI:0.86 ~ 0.90)	0.38 (95% CI:0.35 ~ 0.42)	0.76 (95% CI:0.74 ~ 0.78)	0.59 (95% CI:0.54 ~ 0.63)	4.595% CI:(3.6 ~ 5.6)
Berlin	0.73 (95% CI:0.71 ~ 0.76)	0.96 (95% CI:0.95 ~ 0.97)	0.34 (95% CI:0.30 ~ 0.37)	0.77 (95% CI:0.75 ~ 0.79)	0.80 (95% CI:0.75 ~ 0.84)	12.495% CI:(9.2 ~ 16.6)
STOP	0.67 (95% CI:0.65 ~ 0.69)	0.93 (95% CI:0.92 ~ 0.95)	0.14 (95% CI:0.11 ~ 0.16)	0.71 (95% CI:0.69 ~ 0.73)	0.48 (95% CI:0.41 ~ 0.55)	2.2 (95% CI:1.7 ~ 2.7)
STOPBang	0.72 (95% CI:0.70 ~ 0.74)	0.98 (95% CI:0.98 ~ 0.99)	0.18 (95% CI:0.15 ~ 0.21)	0.73 (95% CI:0.71 ~ 0.75)	0.83 (95% CI:0.77 ~ 0.89)	10.8 (95% CI:7.3 ~ 15.9)
AHI = 15						
GPSS	0.73 (95% CI:0.71 ~ 0.75)	0.60 (95% CI:0.57 ~ 0.63)	0.74 (95% CI:0.72 ~ 0.77)	0.68 (95% CI:0.65 ~ 0.71)	0.6 (95% CI:0.64 ~ 0.69)	4.3 (95% CI:3.6 ~ 5.1)
NoSAS	0.71 (95% CI:0.69 ~ 0.73)	0.59 (95% CI:0.56 ~ 0.62)	0.72 (95% CI:0.69 ~ 0.74)	0.66 (95% CI:0.63 ~ 0.69)	0.65 (95% CI:0.63 ~ 0.68)	3.7 (95% CI:3.1 ~ 4.4)
Berlin	0.70 (95% CI:0.68 ~ 0.72)	0.64 (95% CI:0.61 ~ 0.67)	0.63 (95% CI:0.60 ~ 0.66)	0.62 (95% CI:0.59 ~ 0.65)	0.65 (95% CI:0.62 ~ 0.68)	3.0 (95% CI:2.6 ~ 3.5)
STOP	0.66 (95% CI:0.63 ~ 0.68)	0.40 (95% CI:0.37 ~ 0.43)	0.84 (95% CI:0.81 ~ 0.86)	0.70 (95% CI:0.66 ~ 0.73)	0.60 (95% CI:0.58 ~ 0.63)	3.5 (95% CI:2.8 ~ 4.4)
STOPBang	0.70 (95% CI:0.68 ~ 0.73)	0.69 (95% CI:0.66 ~ 0.72)	0.65 (95% CI:0.62 ~ 0.67)	0.65 (95% CI:0.62 ~ 0.67)	0.69 (95% CI:0.66 ~ 0.72)	4.1 (95% CI:3.5 ~ 4.8)
AHI = 30						
GPSS	0.73 (95% CI:0.71 ~ 0.75)	0.30 (95% CI:0.26 ~ 0.33)	0.90 (95% CI:0.88 ~ 0.91)	0.58 (95% CI:0.53 ~ 0.63)	0.73 (95% CI:0.71 ~ 0.75)	3.8 (95% CI:2.9 ~ 5.1)
NoSAS	0.71 (95% CI:0.68 ~ 0.73)	0.35 (95% CI:0.31 ~ 0.38)	0.88 (95% CI:0.87 ~ 0.90)	0.59 (95% CI:0.54 ~ 0.63)	0.74 (95% CI:0.72 ~ 0.76)	4.0 (95% CI:3.1 ~ 5.1)
Berlin	0.70 (95% CI:0.68 ~ 0.72)	0.29 (95% CI:0.25 ~ 0.32)	0.92 (95% CI:0.90 ~ 0.93)	0.62 (95% CI:0.57 ~ 0.68)	0.73 (95% CI:0.71 ~ 0.75)	4.7 (95% CI:3.5 ~ 6.3)
STOP	0.65 (95% CI:0.63 ~ 0.68)	0.11 (95% CI:0.09 ~ 0.14)	0.97 (95% CI:0.96 ~ 0.98)	0.62 (95% CI:0.53 ~ 0.70)	0.70 (95% CI:0.68 ~ 0.72)	4.0 (95% CI:2.5 ~ 6.4)
STOPBang	0.70 (95% CI:0.68 ~ 0.72)	0.17 (95% CI:0.14 ~ 0.20)	0.95 (95% CI:0.94 ~ 0.97)	0.64 (95% CI:0.57 ~ 0.71)	0.71 (95% CI:0.69 ~ 0.73)	3.9 (95% CI:2.7 ~ 5.6)

**Table 4:** Different AHIs as cut-off points for the diagnosis of obstructive sleep apnea (second group).

Questionnaire	ROC	Sensitivity	Specificity	Positive predictive value	Negative predictive value	Diagnostic odds ratios
AHI = 5						
GPSS	0.77 (95% CI:0.72 ~ 0.83)	0.86 (95% CI:0.78 ~ 0.95)	0.55 (95% CI:0.49 ~ 0.62)	0.31 (95% CI:0.24 ~ 0.38)	0.95 (95% CI:0.91 ~ 0.98)	7.5 (95% CI:4.4 ~ 12.7)
NoSAS	0.76 (95% CI:0.69 ~ 0.82)	0.86 (95% CI:0.78 ~ 0.95)	0.51 (95% CI:0.45 ~ 0.57)	0.29 (95% CI:0.23 ~ 0.36)	0.94 (95% CI:0.90 ~ 0.98)	6.4 (95% CI:3.8 ~ 10.8)
Berlin	0.66 (95% CI:0.59 ~ 0.73)	0.85 (95% CI:0.76 ~ 0.94)	0.38 (95% CI:0.32 ~ 0.44)	0.24 (95% CI:0.19 ~ 0.30)	0.91 (95% CI:0.86 ~ 0.97)	3.5 (95% CI:2.0 ~ 6.0)
STOP	0.67 (95% CI:0.60 ~ 0.74)	0.54 (95% CI:0.42 ~ 0.67)	0.72 (95% CI:0.66 ~ 0.77)	0.31 (95% CI:0.22 ~ 0.40)	0.87 (95% CI:0.82 ~ 0.92)	3.0 (95% CI:1.8 ~ 5.2)
STOPBang	0.72 (95% CI:0.66 ~ 0.79)	0.75 (95% CI:0.63 ~ 0.86)	0.60 (95% CI:0.54 ~ 0.66)	0.31 (95% CI:0.23 ~ 0.38)	0.91 (95% CI:0.87 ~ 0.95)	4.5 (95% CI:2.8 ~ 7.3)
AHI = 15						
GPSS	0.75 (95% CI:0.70 ~ 0.80)	0.62 (95% CI:0.55 ~ 0.70)	0.79 (95% CI:0.72 ~ 0.86)	0.78 (95% CI:0.71 ~ 0.85)	0.64 (95% CI:0.57 ~ 0.71)	6.13 (95% CI:3.41 ~ 11.02)
NoSAS	0.74 (95% CI:0.68 ~ 0.79)	0.75 (95% CI:0.69 ~ 0.82)	0.66 (95% CI:0.59 ~ 0.74)	0.72 (95% CI:0.66 ~ 0.79)	0.70 (95% CI:0.62 ~ 0.78)	5.82 (95% CI:3.57 ~ 9.50)
Berlin	0.66 (95% CI:0.60 ~ 0.72)	0.77 (95% CI:0.71 ~ 0.84)	0.47 (95% CI:0.39 ~ 0.55)	0.63 (95% CI:0.56 ~ 0.70)	0.64 (95% CI:0.55 ~ 0.73)	2.96 (95% CI:1.81 ~ 4.84)
STOP	0.66 (95% CI:0.61 ~ 0.72)	0.77 (95% CI:0.70 ~ 0.83)	0.48 (95% CI:0.40 ~ 0.56)	0.63 (95% CI:0.57 ~ 0.70)	0.64 (95% CI:0.55 ~ 0.73)	3.09 (95% CI:1.89 ~ 5.05)
STOPBang	0.71 (95% CI:0.66 ~ 0.77)	0.63 (95% CI:0.56 ~ 0.70)	0.73 (95% CI:0.65 ~ 0.80)	0.73 (95% CI:0.66 ~ 0.80)	0.63 (95% CI:0.55 ~ 0.70)	4.63 (95% CI:2.68 ~ 8.00)
AHI = 30						
GPSS	0.69 (95% CI:0.62 ~ 0.75)	0.49 (95% CI:0.43 ~ 0.56)	0.80 (95% CI:0.70 ~ 0.90)	0.90 (95% CI:0.85 ~ 0.95)	0.30 (95% CI:0.23 ~ 0.36)	3.84 (95% CI:2.14 ~ 6.90)
NoSAS	0.68 (95% CI:0.61 ~ 0.75)	0.62 (95% CI:0.56 ~ 0.69)	0.68 (95% CI:0.56 ~ 0.79)	0.88 (95% CI:0.83 ~ 0.93)	0.32 (95% CI:0.24 ~ 0.40)	3.47 (95% CI:2.06 ~ 5.85)
Berlin	0.68 (95% CI:0.61 ~ 0.76)	0.73 (95% CI:0.68 ~ 0.79)	0.62 (95% CI:0.50 ~ 0.73)	0.88 (95% CI:0.83 ~ 0.92)	0.38 (95% CI:0.29 ~ 0.47)	4.42(95% CI:2.76 ~ 7.08)
STOP	0.70 (95% CI:0.62 ~ 0.77)	0.73 (95% CI:0.67 ~ 0.78)	0.63 (95% CI:0.51 ~ 0.75)	0.88 (95% CI:0.84 ~ 0.93)	0.38 (95% CI:0.29 ~ 0.47)	4.61 (95% CI:2.88 ~ 7.38)
STOPBang	0.71 (95% CI:0.64 ~ 0.78)	0.82 (95% CI:0.78 ~ 0.87)	0.51 (95% CI:0.39 ~ 0.63)	0.86 (95% CI:0.82 ~ 0.91)	0.43 (95% CI:0.32 ~ 0.55)	4.74 (95% CI:3.02 ~ 7.44)

## 4 Discussion

OSA is a common disorder that leads to sleep fragmentation, decreased arterial oxygen saturation, and poor sleep quality (7). It is associated with several medical disciplines, including respiratory, cardiovascular, endocrine, and ear, nose, and throat (20–22). The high number of complications in patients with OSAS is particularly likely to cause cardiac, cerebral, and renal organ pathologies, leading to an increased risk of cardiovascular and cerebrovascular accidents. These factors and their consequences have made OSA an economic and social burden that has received increasing attention (23, 24). However, due to a lack of awareness, the disease remains underdiagnosed and untreated. It is estimated that nearly 80% of men and 93% of women with moderate to severe sleep apnea go undiagnosed (25), and undiagnosed OSA can lead to multiple problems. Effective early screening is essential to mitigate the negative effects of OSA. Screening tools are used to differentiate patients at high risk of OSA from those at low risk, and they should be easy to use with high sensitivity and acceptable specificity. To date, most OSA screening tools have been validated in patients referred to sleep clinics or sleep laboratories. Screening models based on various combinations of seven factors, including apnea, snoring, observed apnea, BMI, age, sex, and hypertension, have been developed and validated in patients from sleep centers (26–33). These screening scales include NoSAS, Berlin, STOP, and STOP-Bang. The GPSS questionnaire, a recently developed screening tool for OSA in Australia (13), has not yet been validated in other populations. The higher prevalence in some countries, including China, may stem from ethnic and genetic differences—these differences make certain anatomical traits, such as a narrowed airway, more likely to trigger OSA (34). O’Connor et al. showed that craniofacial structural abnormalities, such as a small mandible, were common in Asians, whereas Caucasians more frequently presented with marked obesity (35). Therefore, we validated the performance of the newly developed questionnaire, GPSS, and four commonly used OSA screening questionnaires (NoSAS, Berlin, STOP, and STOP-Bang) in identifying different severity levels of OSA in the Chinese population to assess their applicability in clinical practice.

This study included 2,169 patients in the first group and 310 patients in the second group. The prevalence of OSA was 69.3% in the first group and 79.5% in the second group. The GPSS showed that the first group had a higher specificity and positive predictive value in all OSA patients, with fewer false positives and a lower rate of misdiagnosis. This may be attributed to the fact that Guangzhou, as a capital city, has a population with higher health awareness, making individuals more likely to seek medical attention even for mild symptoms. The second group had a sensitivity, positive predictive value, and negative predictive value in all OSA patients, with fewer missed diagnoses, mainly because Zhanjiang has a relatively worse economy compared to Guang and slightly lower health awareness among the population, who tend to seek specialist care at the hospital only when they have more comorbidities and more typical symptoms. In patients with moderate severe OSA, the first group of patients had lower sensitivity, specificity, positive predictive value, and negative predictive value in all indicators compared to the other four questionnaires while the second group of patients, although having better specificity and positive predictive value than the other questionnaires, had a lower AUC value than the STOP and STOP-Bang questionnaires, thus had lower effect in moderate to severe OSA and should not be used alone in this scenario.

In the comparison of general data, it was shown that there were significant differences in neck circumference, waist circumference, pulse, systolic blood pressure, diastolic blood pressure, age, height, weight, BMI, and sex characteristics between the OSA group and the non-OSA group. It can be seen that OSA patients have the characteristics of a large neck circumference, a large waist circumference, high weight, and high blood pressure. At the same time, previous studies have also shown the above view. Busetto et al. found that BMI was positively correlated with AHI, primarily among overweight and obese individuals. No significant correlation was observed in individuals with normal weight, suggesting that a normal weight does not increase the risk of OSA (36). Additionally, the high triglyceride-waist circumference phenotype has been identified as a risk factor for OSA (37). Whittle et al. used magnetic resonance imaging (MRI) to compare the distribution of soft tissue and fat in the necks of men and women. They found that men had a significantly larger volume of soft tissue in the neck and greater fat accumulation in the palatopharyngeal plane than women. These findings suggest that neck circumference plays an important role in the onset of OSA and may partly explain the higher prevalence of OSA in men than in women (38). OSA is a disease dominated by men, which is not only reflected in the higher prevalence of men than women, but also the 2**–**5 times higher prevalence of OSA in men than women in the community population, while the ratio of clinical visits reaches 8**–**10 times. It is worth noting that men with OSA are more serious than women with OSA, which is manifested by higher AHI, longer apnea events, and more severe SpO2 decline (39–42). This is consistent with the results found in our study, which showed that there were more male patients than female patients in the OSA patients and that the gap between men and women was more obvious in severe OSA patients.

The study suggests that when the GPSS score was ≥8, the sensitivity was 88.6% for all OSA and 93.7% for severe OSA. The specificity and PPV for all OSA gradually increased as the score increased from 8 to 12. In the comparison of sensitivity, specificity, PPV, and NPV of the five questionnaires in patients among the patients from the first group (Table 3), at an AHI threshold of 5, the GPSS significantly outperformed the STOP and STOP-Bang in ROC, Specificity, PPV, and NPV, but showed poorer sensitivity. At an AHI threshold of 15, the GPSS significantly outperformed all questionnaires via ROC, showed poorer sensitivity compared to the STOP-Bang and Berlin, and greater sensitivity than the STOP; showed poorer specificity than the STOP but greater specificity than the STOP-Bang and Berlin; and for the PPV and NPV showed equal values with the NoSAS, STOP-Bang, and Berlin, which were all significantly greater than the STOP. At an AHI threshold of 30, the GPSS significantly outperformed all questionnaires via ROC, showed poorer sensitivity compared to the NoSAS, and greater sensitivity than the STOP and STOP-Bang; showed poorer specificity than the STOP but greater specificity than the STOP-Bang and Berlin; for the PPV, there was no significant difference between any scores; and for the NPV, the GPSS showed equal values with the NoSAS, STOP-Bang, and Berlin, which were all significantly greater than the STOP. In the comparison of sensitivity, specificity, PPV and NPV of the five questionnaires in patients in the (Table 4), at an AHI threshold of 5, the GPSS significantly outperformed the Berlin, STOP and STOP-Bang via ROC; showed equal sensitivity with the NoSAS and Berlin and significantly greater sensitivity than the STOP and STOP-Bang; showed poorer specificity than the STOP and STOP-Bang but greater specificity than the Berlin; for the PPV there was no significant difference between any scores; and for the NPV the GPSS showed equal values with the NoSAS, STOP-Bang and Berlin which were all significantly greater than the STOP. At an AHI threshold of 15, the GPSS significantly outperformed the STOP and Berlin via ROC, showed poorer sensitivity compared to the NoSAS, Berlin, and STOP; showed greater specificity than the NoSAS, Berlin, and STOP; for the PPV, the GPSS was equal to the NoSAS and STOP-Bang but greater than the STOP and Berlin; and for the NPV, there was no significant difference between any scores. At an AHI threshold of 30, there was no significant difference between any questionnaires via ROC; the GPSS showed significantly poorer sensitivity than all other questionnaires but significantly greater specificity; in the PPV there was no significant difference between any questionnaires, while in the NPV, the STOP-Bang showed a greater score than the GPSS, but there was no difference between the GPSS and other questionnaires.

The area under the ROC curve not only integrates the 2 indicators of sensitivity and specificity, but also considers every possible boundary value; thus, it can evaluate the diagnostic value of diagnostic tests more objectively, and it has also been used as a recognized standard evaluation index for diagnostic tests (43). According to the ROC results, the GPSS questionnaire was superior to the other four scales in screening for mild-to-moderate OSA, but it still has room for improvement in severe OSA. Therefore, we recommend the GPSS for early screening of OSA, especially in the screening in primary healthcare settings with limited time, and it should be considered for the screening among patients with a high risk of severe OSA.

The Berlin Questionnaire was first introduced at the Primary Care Sleep Conference in Berlin, Germany, in 1996 (19), and has since become one of the most widely used qualitative diagnostic tools for OSA internationally. The first two sections rely on subjective reports from the patient’s family members, while the third section is based on objective indicators. The STOP questionnaire was simplified by Chung et al. (18) based on the Berlin questionnaire and published the STOP-Bang questionnaire in 2008 (44). The STOP-Bang questionnaire incorporated the objective indicators of BMI, age, neck circumference, and gender on the basis of the STOP questionnaire, which made the STOP-Bang questionnaire’s screening ability to be further improved (45, 46). In 2016, Marti-Soler et al. (17) devised the NoSAS score, which consists of five dimensions, namely neck circumference (N), obesity (O), snoring (S), age (A), and sex (S), with a total score of 0–17, with a neck circumference of >40 cm as a score of 4, a BMI of 25- < 30 kg/m^2^ as 3 points or BMI ≥ 30 kg/m^2^ as 5 points, snoring as 2 points, age ≥55 as 4 points, male as 2 points, and NoSAS score ≥8 as high risk and <8 as low risk. The GPSS questionnaire consists of two parts: part A is the patient’s baseline clinical/demographic information, and part B is the signs or symptoms related to OSA. The first two questions in part B assess the nocturnal OSA symptoms; the last two questions assess daytime OSA symptoms; and the last question assesses medical co-morbidities known to be associated with OSA (13). The four questions in part A (age, sex, BMI, neck circumference or collar size) and the first three questions in part B (snoring, daytime tiredness, and apnea during sleep) are consistent with the STOP-Bang questionnaire. However, the GPSS questionnaire adds to the other questionnaires by assessing medical co-morbidities known to be associated with OSA, including hypertension, diabetes, heart disease, or depression. Epidemiological data (47) suggest that OSA is present in 30–50% of patients with hypertension, while 50% of patients with OSA have hypertension, and about 80% of patients with recalcitrant hypertension have OSA. The risk of coexisting OSA is increased by 1.8-fold in patients with type 2 diabetes mellitus compared to non-diabetic patients (48). In 2008, the International Diabetes Federation (IDF) recommended that all patients with type 2 diabetes should be routinely screened for co-morbid OSA (49). Studies have found that depressive symptoms are the most common psychiatric symptoms in patients with OSAHS (50), with an incidence of 7 to 63% (51). It severely affects patients’ quality of life (52) and may also exacerbate the neurological damage in the brain associated with OSA (53). Some existing studies have confirmed that there is a strong association between ischemic heart disease and OSA, and the incidence of OSA in patients with ischemic heart disease ranges from 35 to 40% (54), and OSA can significantly increase the morbidity and mortality of patients with ischemic heart disease, and its risk level is related to the severity level of OSA (55). Thus, it can be seen that hypertension, diabetes, heart disease, or depression are common comorbidities of OSA. Therefore, incorporating an assessment of known comorbidities into screening questionnaires may improve their positive predictive value for identifying OSA.

However, there are some limitations of this study. (1) This study is a retrospective study, which limits the ability to establish a causal relationship. (2) The population is limited to the Chinese population. (3) This study included patients from the sleep medicine centers of two hospitals, and there may be differences in patients seeking medical help for sleep disorders or related diseases due to economic differences between the two places, which may lead to bias in the neck circumference, BMI, and OSA positive rate of the two patient groups, and there is a certain selection and confounding bias. Therefore, further multicenter studies are needed in the future to validate the validity of the GPSS questionnaire.

## 5 Conclusion

According to the ROC results, the GPSS questionnaire outperformed the other four scales in screening for mild-to-moderate OSA but showed limitations in detecting severe OSA. Therefore, we recommend the GPSS for early screening of OSA, especially in primary healthcare settings where time is limited. For patients at high risk of severe OSA, combining the GPSS with other screening tools may enhance diagnostic accuracy.

## Data Availability

The original contributions presented in the study are included in the article/supplementary material, further inquiries can be directed to the corresponding authors.
